# Dental Rehabilitation of a Child Diagnosed With Alopecia Areata Totalis: A Case Report

**DOI:** 10.7759/cureus.106874

**Published:** 2026-04-12

**Authors:** Nathalie Abou Samra, Samia Aboujaoude

**Affiliations:** 1 Pediatric Dentistry, Aman Hospital, Doha, QAT; 2 Pediatric Dentistry and Public Dental Health, Lebanese University, Beirut, LBN

**Keywords:** alopecia areata totalis, autoimmune disease, dental rehabilitation, hair regrowth, oral infection

## Abstract

Alopecia areata totalis is a variant of alopecia areata, a common autoimmune disorder resulting in non-scarring hair loss. Although its pathogenesis remains unclear, both genetic predisposition and environmental triggers have been implicated, of which chronic inflammatory and infectious conditions are considered potential contributors through persistent immune stimulation. This report presents a case of alopecia areata totalis in a five-year-old female patient who presented with extensive untreated dental caries and chronic oral infection. The patient underwent comprehensive dental rehabilitation under general anesthesia, which included elimination of all infectious foci and restoration of oral health. Notably, a marked hair regrowth was observed during follow-up, in the absence of ongoing dermatological treatment, highlighting the possible association between chronic oral infection and alopecia areata. This case highlights the potential role of chronic oral infection as a contributing environmental trigger in alopecia areata and emphasizes the importance of a multidisciplinary approach in the management of pediatric patients with autoimmune conditions.

## Introduction

Alopecia areata totalis is a variant of alopecia areata (AA), a common hair loss disorder, consisting of well-demarcated round or oval non-scarring hair loss patches [1]. The extent and pattern of hair loss may vary among individuals. In approximately 1-2% of AA cases, the condition may evolve from initial small, localized patches to more extensive clinical forms. Disease progression can result in alopecia totalis, characterized by complete loss of the scalp hair, or may further advance to alopecia universalis, defined by complete loss of both scalp and body hair [2]. Typically, the scalp is involved up to (90%) with subsequent loss of eyebrows and eyelashes. In advanced cases, AA may extend to the beard, extremities, and trunk.

As it affects highly visible areas, the condition can have a significant emotional impact on children, leading to decreased self-esteem, emotional distress, and social withdrawal. AA is also associated with increased susceptibility to bullying, reduced quality of life, and development of psychiatric comorbidities [3]. Moreover, female patients have been reported to be more negatively affected in terms of self-acceptance and confidence [4]. In children, the prevalence is estimated 1.92%, compared to 1.47% in adults [5]. According to Dan et al. (2007), AA affects males and females equally [6]. Other studies advocate a higher incidence rate in the female population compared to males [7].

In addition to genetic predisposition, several factors contribute to the etiology of AA, including immunological, environmental, and possibly psychological triggers. In affected patients, a defect in hair follicle immune privilege is suspected, leading to the activation of natural killer cells and T cells involved in the inhibition of normal hair growth [8]. Recent evidence indicates that cytotoxic CD8⁺NKG2D⁺ T cells are the primary effector cells in the pathogenesis of alopecia areata, directly infiltrating hair follicles and driving autoimmune destruction through production of interferon‑γ and other pro‑inflammatory signals; while NK cells also express NKG2D, their role appears secondary compared to the dominant pathogenic contribution of these autoreactive CD8⁺ T cells [9].

AA is an autoimmune disorder marked by a sudden interruption of the normal hair cycle, where follicles prematurely shift from the growth (anagen) stage into the shedding (telogen) stage [10]. This process results from immune dysregulation and inflammatory response following the breakdown of the hair follicle’s immune privilege. With the loss of this protective privilege, the immune system identifies follicular proteins as foreign, which initiates a cell-mediated immune response. This misidentification leads to the recruitment of T lymphocytes around the follicle bulb, where they release cytokines and inflammatory mediators that disrupt normal follicular activity and suppress hair production.

Among the cytokines involved, interferon-gamma (IFN-γ) is considered an important cytokine in maintaining immune activation. Additional molecules, including interleukin (IL)-15 and IL-2, enhance the process by stimulating immune cell survival and activation. The combined action of these mediators is transmitted through the Janus kinase/signal transducer and activator of transcription (JAK-STAT) pathway, whose dysregulation sustains chronic inflammation and follicular damage. Despite inflammation disrupting follicular activity, the epithelial stem cell reservoir remains intact, allowing hair regeneration once the immune response is controlled. Targeted therapies, such as JAK inhibitors, reduce inflammation and promote follicular recovery in affected patients [8,10].

Other factors, such as vaccinations and viral infections, together with certain types of hepatitis, can potentially trigger alopecia. Although a potential link with focal infection of dental origin has been suggested, only a limited number of cases have been reported, particularly in children [3].

This report describes the case of a five-year-old female child presenting with AA affecting the scalp, eyelashes, and eyebrows, who subsequently showed notable hair regrowth following comprehensive dental rehabilitation. 

## Case presentation

**Figure 1:**
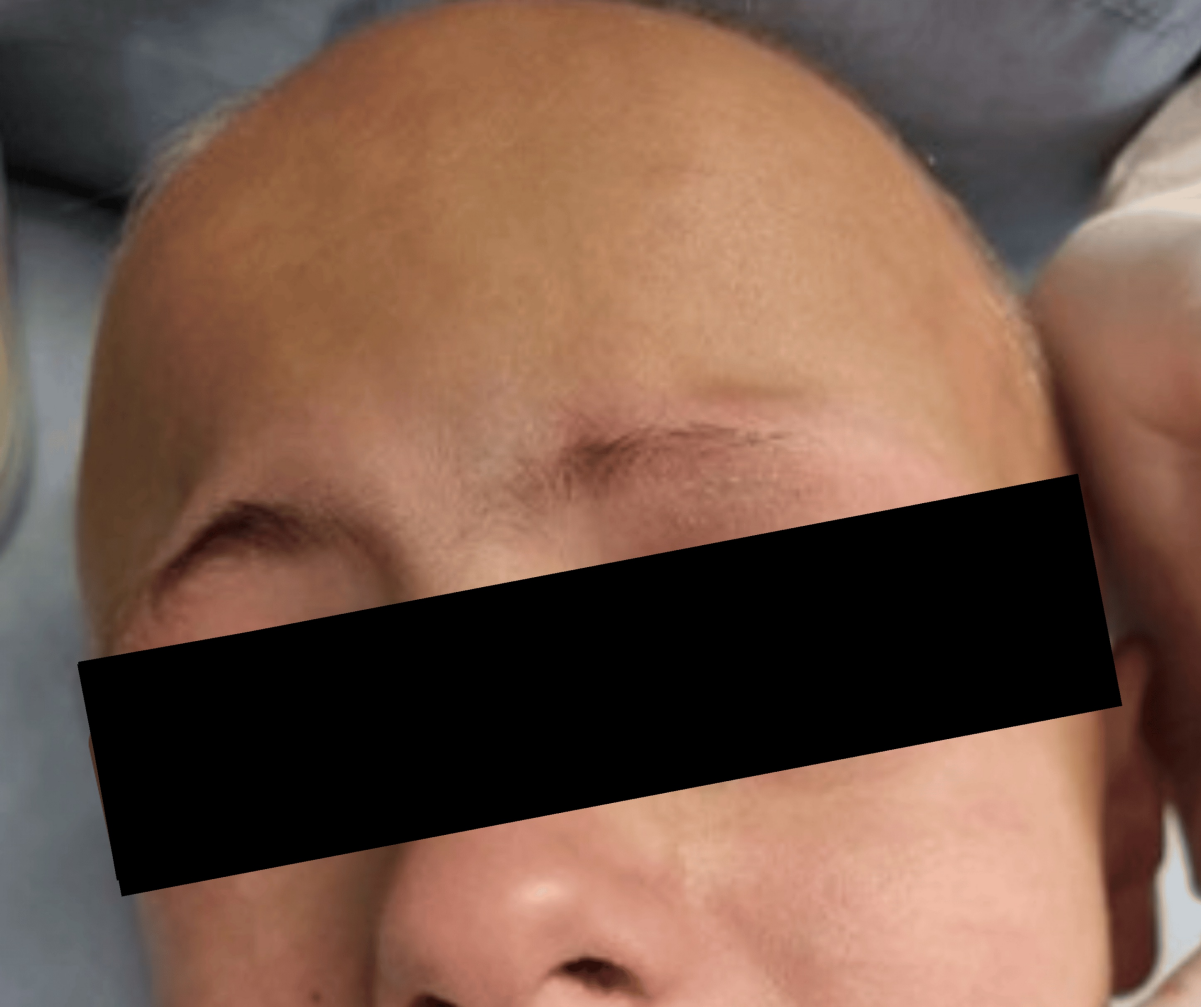
Five-year-old girl presenting with complete scalp hair loss consistent with alopecia areata totalis

A five-year-old girl presented to our pediatric dental clinic accompanied by her parents, who were concerned about her dental appearance and impaired chewing function. The extraoral examination revealed extensive hair loss; the scalp appeared completely devoid of hair with a smooth, soft surface. Intact skin texture with no apparent lesions, abnormalities, or signs of inflammation or erythema was noticed. Eyebrows and eyelashes were present but appeared to be sparse (Figure 1).

Medical history showed that the child was born prematurely at 34 weeks and admitted to the neonatal intensive care unit for two weeks. At birth, the parents reported that she had normal scalp hair in terms of quantity and distribution. At the age of four, she was diagnosed by her dermatologist with AA totalis after a sudden onset of patchy hair loss. A familial predisposition to alopecia was noted, as the patient’s father has a history of patchy AA, with no history of systemic disease or known allergies. Systemic prednisolone and iron supplementation were prescribed at that time but were discontinued prior to dental treatment.

**Figure 2:**
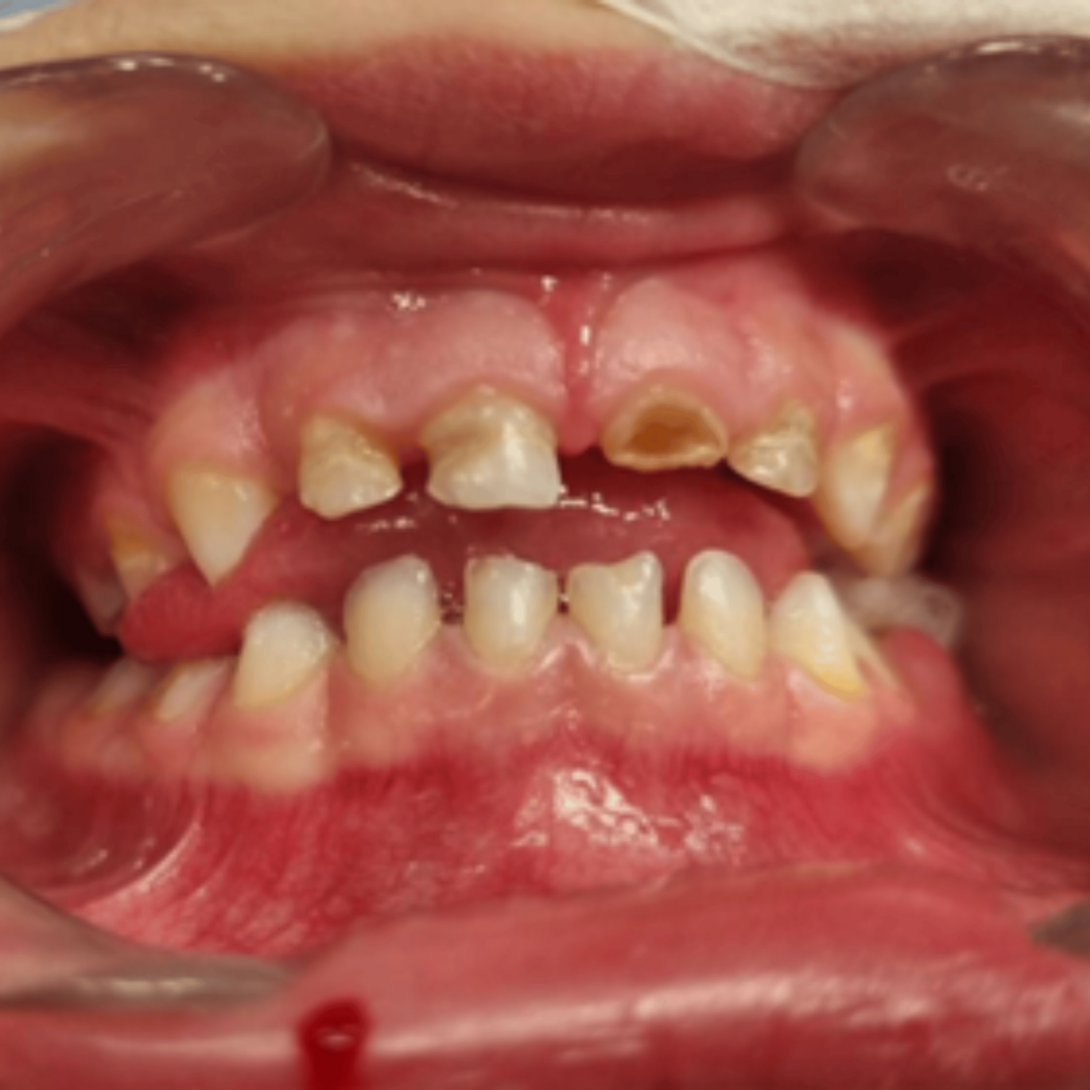
Preoperative photograph showing severe early childhood caries

**Figure 3:**
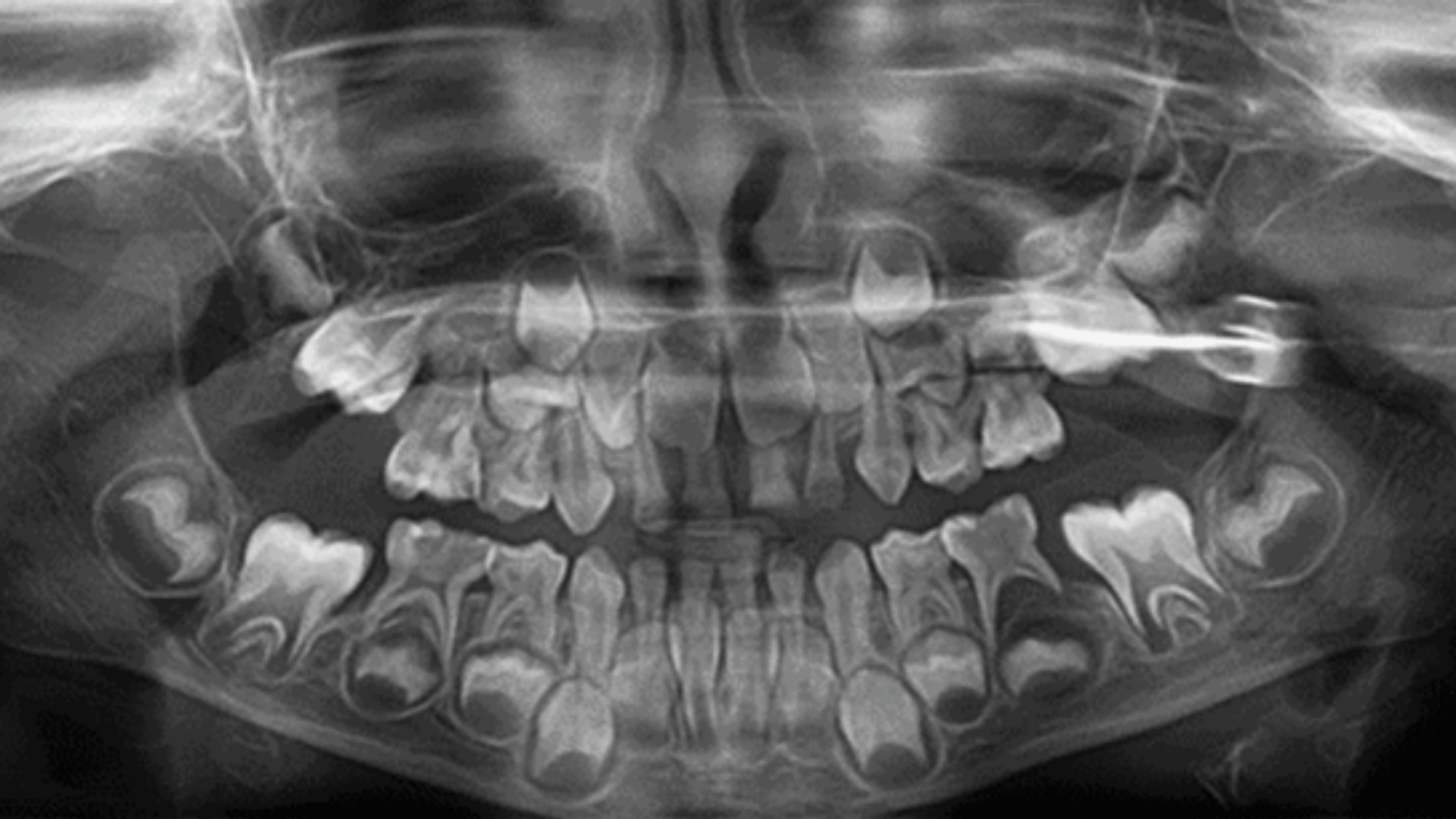
Preoperative panoramic radiograph showing generalized early childhood caries, teeth with advanced pulpal pathology, and radiographic signs of chronic oral infection associated with the second left lower primary molar.

Intraoral examination revealed chapped lips with normal soft tissues. On palpation, no regional lymphadenopathy was noticed. Multiple dental caries with a gingival abscess on the left mandibular second primary molar were observed. Clinical examination and panoramic radiography revealed dental caries affecting both the maxillary and mandibular primary teeth. The maxillary central incisors (#51, #61) showed extensive coronal destruction, while the lateral incisors and canines (#52, #53, #62, #63) had carious lesions limited to dentin. The maxillary and mandibular right first and second primary molars (#54, #55, #84, #85) demonstrated extensive caries with pulpal involvement. Caries without pulpal involvement were noted on the mandibular left first primary molar (#74) and maxillary left second primary molar (#65). The mandibular left second primary molar (#75) also showed pulpal involvement with periapical radiolucency secondary to pulpal infection. Overall, the patient presented with severe early childhood caries requiring comprehensive dental rehabilitation (Figures 2-3).

Due to negative behavior (Frankl score I) with marked uncooperativeness and anxiety, the patient was unable to tolerate dental treatment, and full-mouth rehabilitation was therefore completed under general anesthesia. Pulpotomies and placement of stainless-steel crowns were performed on the maxillary and mandibular right first and second primary molars, as well as the maxillary and mandibular left first primary molars. Additionally, the mandibular left second primary molar and both maxillary central incisors were extracted. The maxillary lateral incisors were restored using composite resin.

**Figure 4:**
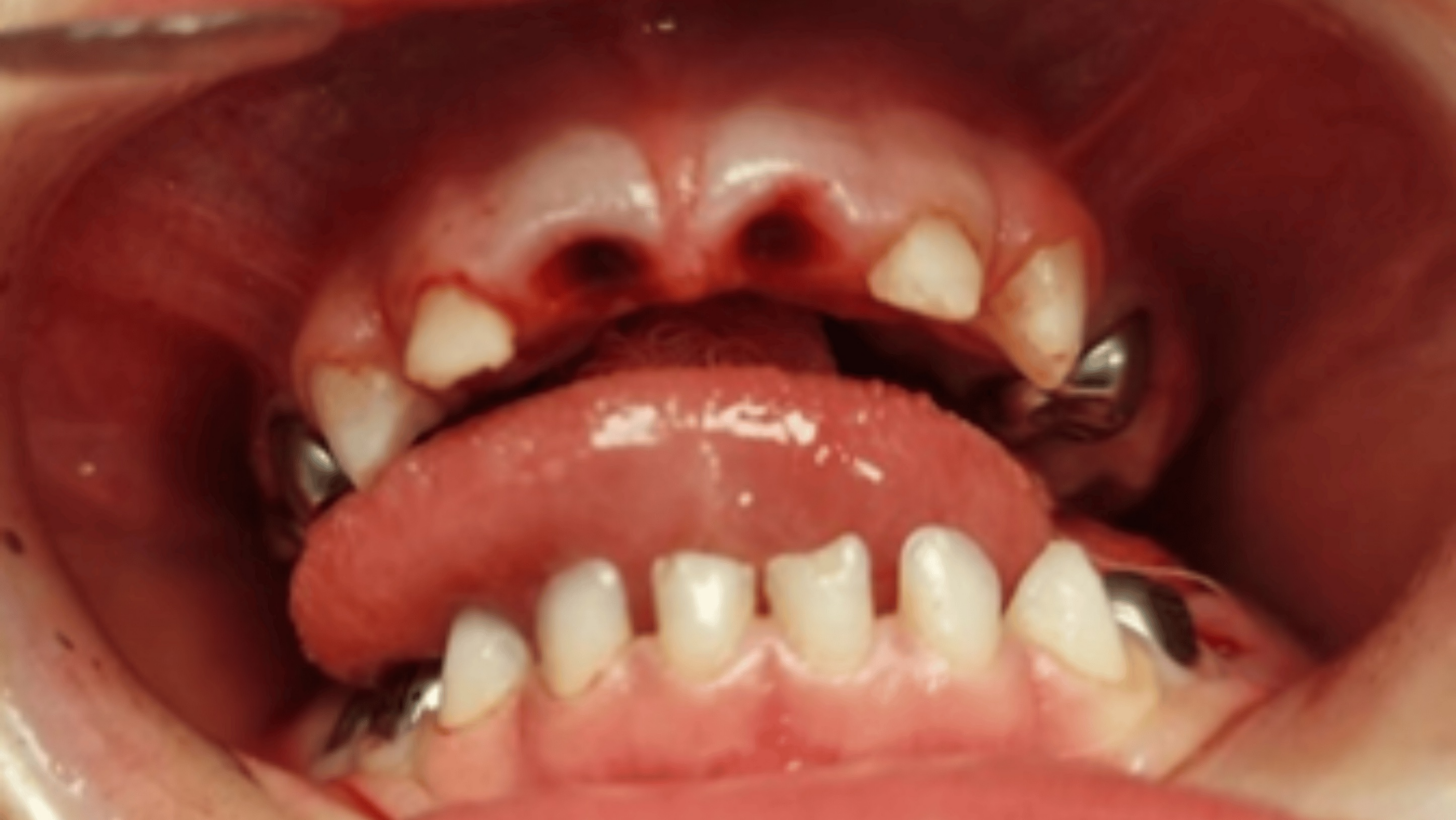
Post-operative clinical photograph illustrating improved oral health status following elimination of chronic dental infection and successful placement of stainless steel crowns after pulp therapy.

The patient was discharged from the hospital on the same day with a prescription of paracetamol (Dafalgan® syrup 100 mg/5 ml) 10 ml every six hours, to reduce pain for three days. An antibiotic therapy (Ospamox® 250 mg/5 ml, 25 mg/kg/day) was recommended for seven days to prevent post-procedural infections. The patient weighed 17 kg at the time of treatment, and all medications were dosed accordingly. Figure 4 shows the postoperative improved oral health.

**Figure 5:**
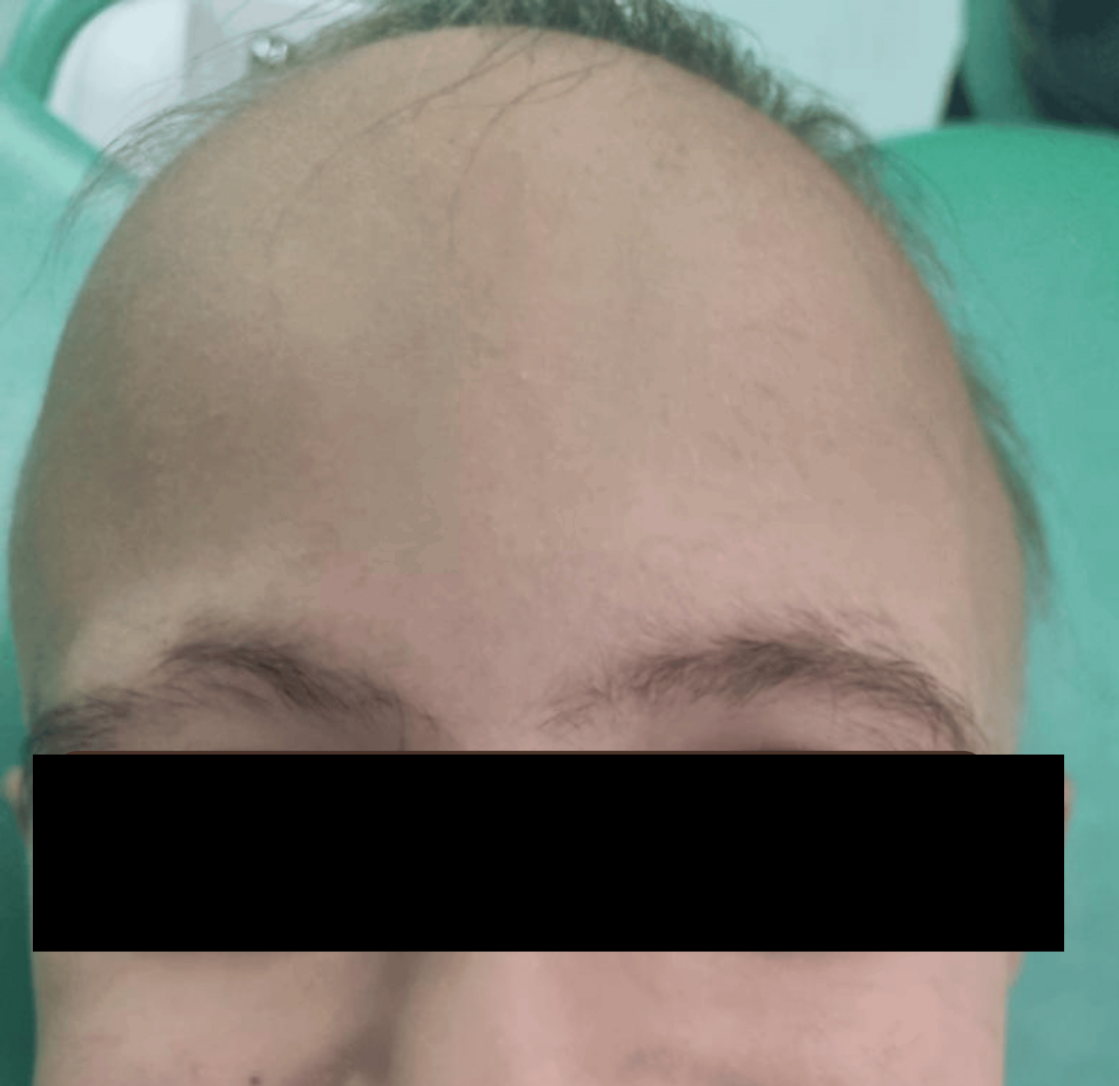
Clinical photograph showing scalp hair status with observable hair regrowth at the two-month follow-up.

At the one-week recall, a Gropper appliance was placed to restore both esthetics and function following the extraction of the maxillary central incisors. At the two-month follow-up, noticeable hair growth, including eyebrows and eyelashes, was noticed (Figure 5).

## Discussion

In the present case, significant hair regrowth with newly emerging hair shafts was noted following comprehensive dental rehabilitation. This temporal association suggests a possible relationship between the elimination of chronic oral infection and the reactivation of hair follicle activity. It highlights the potential association between oral health conditions and alopecia, an area that remains poorly documented in the literature.

AA is considered a complex autoimmune disorder with a genetic basis, characterized by an immune attack against actively growing (anagen) hair follicles. A previous report suggests that chronic oral infections, areas of chronic inflammation, are more likely to occur in children with an underlying genetic predisposition for AA [11]. In such cases, the persistent local inflammatory activity could contribute to systemic immune dysregulation, ultimately exacerbating hair follicle damage. AA has also been associated with the presence of circulating autoantibodies, reflecting its autoimmune nature. The latter antibodies include both hair follicle-specific antibodies as well as non-organ-specific autoantibodies (e.g., antinuclear antibodies, thyroid autoantibodies) [12].

Agrawal et al. suggest that oral infections are known to induce the release of pro-inflammatory cytokines, such as IL-6, tumor necrosis factor-α (TNF-α), and IL-1β, into the systemic circulation [13]. These mediators can disrupt the immune privilege of hair follicles, activate autoreactive T cells, and, in genetically predisposed individuals, trigger or worsen hair loss [14,15].

According to Kaur et al., chronic dental infections, including chronic periodontitis, periapical abscesses, or infected root canals, may trigger a sustained immune response accompanied by the release of circulating antibodies [16]. Although these antibodies are initially directed against microbial antigens, cross-reactivity with host tissues may occur through mechanisms such as molecular mimicry, whereby certain oral bacterial components share structural similarities with human proteins. In individuals with genetic susceptibility, this aberrant immune response could contribute to the systemic autoimmunity observed in AA, thereby establishing a potential link between oral pathology and hair follicle damage [13].

Alternatively, some studies propose a different mechanism linking dental pathology to AA, based on a trigeminal-sympathetic reflex [3]. In this model, a remote mechanical or infectious stimulus triggers a centripetal response via a triple-neuron pathway. The subsequent centrifugal conduction, mediated by the sympathetic nucleus near the terminal branches of the trigeminal nerve, may induce vasoconstriction of the pilosebaceous unit. This reduction in blood flow can lead to structural and functional alterations within the hair follicle, ultimately contributing to hair loss.

## Conclusions

This case illustrates an atypical and severe presentation of alopecia. Although dental infections may not directly cause AA, they may act as potential triggers or aggravating factors by maintaining systemic inflammation and promoting immune activation. In patients with unexplained or recurrent AA, assessing and managing potential oral sources of infection should be considered as part of a comprehensive, holistic treatment approach.
